# Taxonomy of the form and function of primary care services in or alongside emergency departments: concepts paper

**DOI:** 10.1136/emermed-2018-208305

**Published:** 2019-09-07

**Authors:** Alison Cooper, Michelle Edwards, Janet Brandling, Andrew Carson-Stevens, Matthew Cooke, Freya Davies, Thomas Hughes, Katherine Morton, Aloysius Siriwardena, Sarah Voss, Jonathan Benger, Adrian Edwards

**Affiliations:** 1 Division of Population Medicine, School of Medicine, Cardiff University, UK; 2 Faculty of Health and Applied Sciences, The University of the West of England, Bristol, UK; 3 Warwick Medical School, University of Warwick, Coventry, West Midlands, UK; 4 Emergency Department, John Radcliffe Hospital, Oxford, Oxfordshire, UK; 5 Lincoln School of Health and Social Care, University of Lincoln, Lincoln, Lincolnshire, UK

**Keywords:** emergency departments, primary care, emergency care systems, primary care

## Abstract

Primary care services in or alongside emergency departments look and function differently and are described using inconsistent terminology. Research to determine effectiveness of these models is hampered by outdated classification systems, limiting the opportunity for data synthesis to draw conclusions and inform decision-making and policy. We used findings from a literature review, a national survey of Type 1 emergency departments in England and Wales, staff interviews, other routine data sources and discussions from two stakeholder events to inform the taxonomy. We categorised the forms inside or outside the emergency department: inside primary care services may be integrated with emergency department patient flow or may run parallel to that activity; outside services may be offered on site or off site. We then describe a conceptual spectrum of integration: identifying constructs that influence how the services function—from being closer to an emergency medicine service or to usual primary care. This taxonomy provides a basis for future evaluation of service models that will comprise the evidence base to inform policy-making in this domain. Commissioners and service providers can consider these constructs in characterising and designing services depending on local circumstances and context.

## Introduction

Worldwide, increasing pressure on emergency departments from rising demand,^1^ has led to much interest in different models of service delivery, including the use of primary care services in or alongside emergency departments.^2–4^ However, the way these primary care services look and operate varies depending on local context and whether they are required to operate closer to an emergency medicine service or to usual primary care. Research to evaluate the effectiveness of different service models (including patient experience, service and cost-effectiveness outcomes) is hampered by inconsistent terminology, outdated taxonomies and heterogeneous, single-site study designs. This limits the opportunity for data synthesis to draw conclusions that will inform decision-making and policy.^5–7^ Research is urgently needed to understand if the form these services take supports the intended function,^8^ and requires an updated taxonomy to enable comparison of models and outcomes.

The UK has a universal healthcare system, the National Health Service (NHS), funded though taxation.^9^ Primary care is led by general practitioners (GPs), community-based doctors with generalist training, supported by nurses, nurse practitioners and allied health professionals, often with additional diagnostic and prescribing skills working as independent clinicians. Urgent and emergency healthcare services are varied and described using interchangeable terminology (table 1). Three main GP roles are described for primary care services associated with emergency departments (table 1): treating patients identified as having primary care type problems in a unit alongside the emergency department including walk-in centres, urgent care centres or traditional out-of-hours services; screening patients at the front door of the emergency department to redirect those with primary care type problems to an alternative service off site; or fully integrated with the emergency department service, treating patients presenting with a wider range of conditions.^10^ Identification of patients for these services is also varied, with triage (a clinical activity to sort patients by acuity so that those with the greatest need are seen first) and streaming (an operational activity to sort low acuity patients by clinician availability and suitability) sometimes combined or as separate activities. Embedded and co-located  are further terms that have been used to describe primary care models, where clinicians receive patients streamed from the emergency department. (table 1)^11^

**Table 1:** UK urgent and emergency healthcare services^10–12^

Emergency department	Hospital-based ‘front door’ departments for patients with accidents or emergencies.
Minor injuries unit	Care for minor injuries only; may be nurse led.
Walk-in centre	Walk-in access for unscheduled urgent care. May include minor injuries and minor illness; may be nurse led.
Urgent care centre	Unscheduled care for minor injuries and minor illness. Includes minor injury units and walk-in centres; may be nurse led.
Urgent treatment centre	GP-led urgent care centres.
GP in-hours	GP-led primary care services between 8:00 and 18:30 hours.
GP out-of-hours	GP-led services available out-of-hours (18:30-8:00 hours) and weekends, not usually receiving referrals directly from the emergency department.
Alongside the emergency department	GP services located alongside or next to the emergency department.
Screening at the emergency department front door	GPs working at the front of the department screening attendees and either treating or diverting to other places - effectively acting as a filter.
Fully integrated with the emergency department	GP services fully integrated into a joint operation covering the whole range of unscheduled primary care and emergency services.
Embedded into the emergency department	GPs working within the emergency department alongside emergency clinicians, receiving patients streamed as appropriate for primary care.
Co-located urgent care centre	GPs working in a separate area next to the emergency department, receiving patients who have been advised to attend through telephone assessment service (eg, ‘National Health Service 111’) or streamed via the emergency department nurse.

GP, General Practitioner.

NHS England adopted a policy (2017) where emergency departments could apply for capital bid funding (one-off payments) to implement new or develop existing services to support GP streaming.^3^ This has changed the nature of emergency department services and how they function, with evolving relationships with primary care services and the sorting of patients depending on patient acuity and clinician availability. Language to describe the different services is used inconsistently, with considerable ambiguity around the term ‘co-located’. Also, GPs rarely perform a screening role at the emergency department front door. Agreed and consistent terminology is needed to describe the form these services now take, and if form supports the intended function, so that we can understand which service models are being implemented and how they work. The terminology also needs to reflect the current developments in primary care provision, with a broader range of staff than GPs alone.

Recognising this evidence gap, in 2015 the UK National Institute for Health Research (NIHR) Health Services and Delivery Research programme commissioned research to evaluate the effectiveness, safety, patient experience and system implications of the differing models of primary care services in or alongside emergency departments. Two research teams were commissioned, led from Cardiff University and the University of the West of England, Bristol.^13 14^ We aimed to jointly develop a taxonomy describing the form and constructs that influence the function of primary care service models in or alongside emergency departments, to provide the framework for further research and comparing effectiveness between service models.

### Obtaining background information

To understand the nature of the various services in existence, we gathered background information from multiple data sources including: a literature review; a national survey of all Type 1 emergency departments in England and Wales; staff telephone interviews; additional NHS data sources; and early selected case site visits.

### Data sources

### Rapid realist literature review

We undertook a rapid realist literature review,^15^ from April to November 2017, developing theories about how GPs and models of primary care services in or alongside emergency departments work in different contexts to explain varying outcomes, that may be useful for policy-makers.^16^ We sourced research papers from earlier systematic reviews, and supplemented them with updated database searches and citation tracking, also creating an expert group from our co-applicants to assist theory development and guide searches. Our theories were developed from 96 articles to explain: how staff interpret the streaming system; different roles GPs adopt in the emergency department setting (traditional GP, extended role GP, gatekeeper or emergency medicine clinician, alongside other primary care staff); and how these factors influence patient (experience and safety) and organisational (demand and cost-effectiveness) outcomes.^16^

### National survey

We developed a survey, administered through online surveys, to capture data about GPs and models of primary care services associated with emergency departments (see online supplementary data 1). The survey topics covered: the geography of the service related to the emergency department; disciplines of the primary care staff providing the service; how and what type of patient groups were selected for the service; use of investigations; funding and governance arrangements; the aims of the service and whether these had been achieved; enablers and barriers to setting up the service and changes made or planned for the future. The design was informed by recent systematic reviews,^5 17 18^ and a similar survey conducted by the Primary Care Foundation in 2010,^10^ with multiple choice questions and additional space for free text comments. We ran a pilot with our co-applicants and local academic GPs, and iterations were made.

10.1136/emermed-2018-208305.supp1 Supplementary file 1

An invitation email to participate in the study was sent to the clinical directors of all Type 1 emergency departments, consultant-led 24 hours services with full resuscitation facilities,^19^ in England (n=171) and Wales (n=13); first contacted 13 September, reminder 27 September 2017. The study was advertised in the Royal College of Emergency Medicine (UK) monthly news bulletin. Co-applicants (Matthew Cooke and Tim Rainer) sent a further follow-up email in October 2017 to non-responders to encourage participation and the survey was kept open until 28 February 2018. Summary data were extracted through online surveys and exported onto a secure database at Cardiff University.

### Staff interviews

We purposively sampled a selection of emergency departments that described variation in services, to gather more in-depth qualitative data. Clinical directors from 20 departments agreed to participate in a 30–60 min audio-taped telephone interview. Questions were tailored, based on their survey responses, and included: how the staff worked; effects on patient demand and flow; meeting the aims of the service and changes; patient safety; and implications for the wider system (see example in online supplementary data 2).

10.1136/emermed-2018-208305.supp2 Supplementary file 2

NHS England also provided the study team with a list of emergency department sites that had applied for capital funding in 2017 to support GP streaming. We contacted the senior responsible officer for the application at each bidding organisation, with 38 agreeing to complete semi-structured telephone interviews (interview guide online supplementary data 3) about how their emergency department currently operates, and their plans for implementing new models of GP services in the emergency department.

10.1136/emermed-2018-208305.supp3 Supplementary file 3

### Additional data sources

Further information to inform the taxonomy was derived from routinely collected data (eg, https://www.nhsbenchmarking.nhs.uk/ and https://www.healthylondon.org/resource/london-uec-stocktake/) and publicly available documents (including Care Quality Commission reports, Board papers and news items sourced from internet searches). Data from 10 selected study sites (five from the Cardiff University project and five from University of West of England) were available to provide further detail about constructs needed in the taxonomy to cover wider system, department and individual level factors. We visited each study site and collected qualitative data through observations and informal or semi-structured audio-taped staff interviews. Because data were collected from multiple sources, we sometimes encountered elements of conflict between these sources. To resolve this, we used a hierarchy approach in which fieldwork observations (where available) were considered the most reliable, followed by clinical director interviews, survey responses and other data sources, in descending order of reliability.

### Ethics committee approvals

The survey and follow-up interviews were categorised as an NHS service evaluation. Ethics review for the survey and follow-up interviews was carried out by Cardiff University School of Medicine Research Ethics Committee and permission was granted on 29/07/2017 (ref 17/45). The interviews with sites that applied for capital funding to support primary care streaming were conducted as research, with approval from the Health Research Authority (HRA: 230848).

### Findings from the survey, interviews and additional data sources

We had 71 English and 6 Welsh survey responses (n=77/184, 42%). Additionally, we obtained data for 41 English departments from additional data sources, including another five English Type 1 departments that had not been invited to complete the survey (status can change year on year), totalling information on 62% (n=118/189) of Type 1 emergency departments in England and Wales (seen in online supplementary data 4). Of our 71 English survey responders, 82% (n=58/71) applied for capital bid funding, and of our 100 non-responders in England, 84% (n=84/100) applied for capital bid funding.

10.1136/emermed-2018-208305.supp4 Supplementary file 4

The data demonstrated the complexity of models in use and inconsistency in the language being used to describe the different services, with considerable ambiguity around the term ‘co-located’. Primary care clinicians associated with emergency departments, separate to traditional GP out-of-hours services, included a mix of GPs, advanced nurse practitioners and nurses working regular or ad hoc shifts in different ways, seeing different patient groups (see online supplementary data 5). No survey responses or information from other sources indicated that the only role GPs had was to screen patients on arrival at the emergency department. A range of characteristics for employment hours, contracting models and IT systems, was described. Access to investigations, the extent of primary care patient demand in the emergency department, practitioner experience and interest in emergency medicine, and the degree to which they were encouraged to use emergency medicine or primary care protocols also varied. Findings showed that existing classification systems for these service models were not adequate to support research and administration going forward. Therefore, an updated taxonomy was necessary to provide a framework for further research and enable comparison of models and outcomes.

### Formulation of the taxonomy

We consulted with stakeholders for assistance in how to focus the taxonomy and classify the service models in a way that would be useful for commissioners, policy-makers, practitioners, researchers and service users. Our initial stakeholder conference was held in Bristol in February 2018. We invited survey respondents and key authors from the literature. We also used contacts from the research groups to recruit leaders from the Royal Colleges of Emergency Medicine and General Practitioners, NHS Improvement, Care Quality Commission and patient and public contributors. Participant groups included: commissioners and policy-makers (n=6); clinical leads and emergency department clinicians (n=8); GPs and nurse practitioners (n=6); public and patient representatives (n=8); and research team members (n=14). We seated our stakeholders in these separate groups in order to capture different perspectives, with a research team member facilitating each table discussion using structured guidance.

We developed a glossary of terms potentially useful for characterising the services from the rapid realist review, survey and interviews and circulated this in advance (see online supplementary file 6). This and a summary of the findings from the survey, review and interviews were presented to provide a platform from which to initiate group discussions. There were two structured workshops; the first about why participants would find a taxonomy important, and the second about priorities for classifying models. Facilitators gave group feedback to the plenary discussions. Data were captured through flip charts and note taking from research team members (Nigel Pearson and Delyth Price).

10.1136/emermed-2018-208305.supp6 Supplementary file 6

After reflection and discussion, participants agreed that a taxonomy was needed to adequately describe and define this complex system, to support evaluation and to guide policy decisions. An important conclusion was that it should describe both the structure—the form—and constructs that influence the function, that is whether the service operates closer to an emergency medicine service or to usual primary care service provision. A key conceptual underpinning was that although taxonomies tend to present mutually exclusive categories of models, in the case of emergency department primary care models, stakeholders viewed it as a spectrum of integration, from highly integrated with the emergency medicine service to more separate primary care service models, often without clear distinction in practice.

### The FORM of primary care service models in or alongside emergency departments

**Figure 1:**
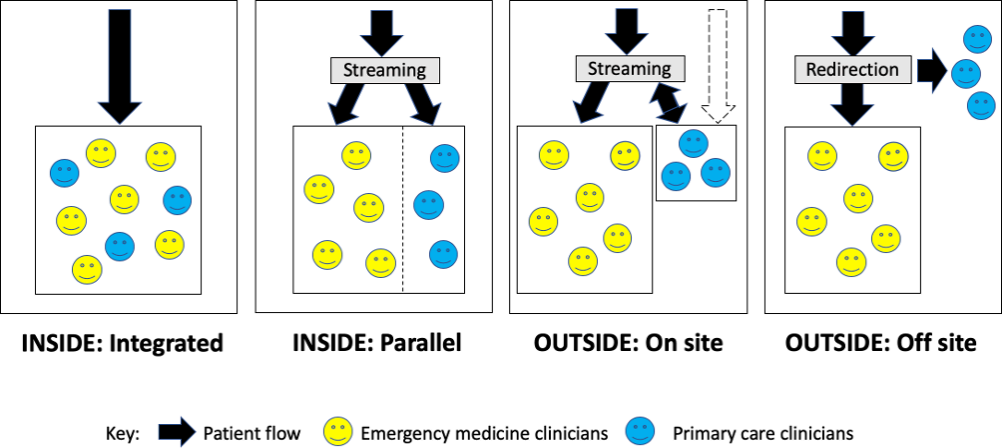
The form of primary care service models in or alongside emergency departments.

Project team members (AE, AC, ME, NP, DP, SV, KM and JB) met in June 2018 to discuss learning and feedback from the event, and from the site visits that had since taken place, to map the taxonomy structure, its labelling and definitions. Location of the service, INSIDE or OUTSIDE the emergency department, was proposed as a useful classification of form—reflecting the patient’s journey and experience, and often aligning with staff contractual arrangements, governance responsibility and accountability (figure 1, definitions in table 2). The INSIDE models varied from those in which primary care clinicians  are integrated with emergency medicine staff or in which they work in a separate parallel primary care service. An alternative primary care service OUTSIDE the emergency department could be on the same hospital site—which we termed on site—or elsewhere, which we termed off site*.

**Table 2:** Taxonomy to describe the form of primary care service models in or alongside emergency departments

INSIDE the emergency department	Patients access a primary care service within the emergency department.
INSIDE: integrated	The primary care service is fully integrated with the emergency medicine service.
INSIDE: parallel	There is a separate primary care service within the emergency department, for patients with primary care type problems.
OUTSIDE the emergency department	Patients access a primary care service separate to the emergency department.
OUTSIDE: on site	The primary care service is elsewhere on the hospital site.
OUTSIDE: off site	The primary care service is off site (may include telephone advice via 111, or pharmacies, dentists, opticians, urgent care centres or registered in-hours or out-of-hours primary care services)*.

*These services are distinct from emergency department provision so are not represented further in the taxonomy.

### Conceptual spectrum of integration: constructs that influence the FUNCTION of these services

**Figure 2:**
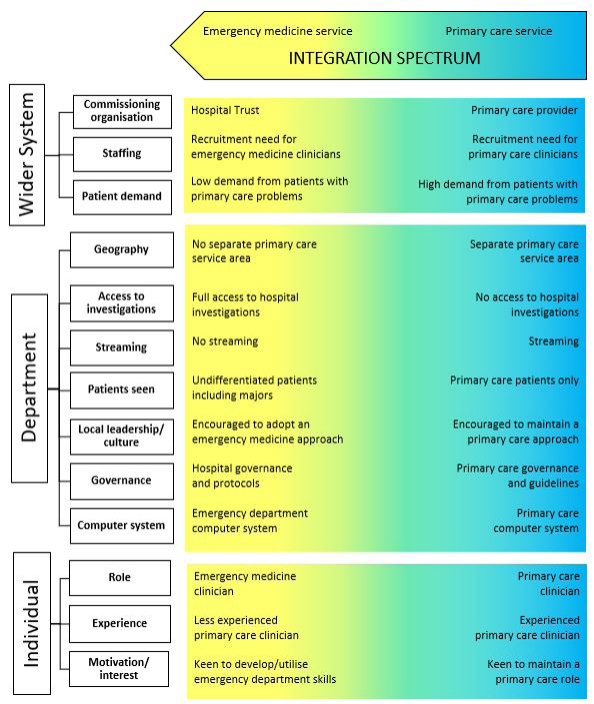
Conceptual model identifying constructs that influence the function of primary care services in or alongside emergency departments

Classifying services simply by form, however, did not always represent the function of these services—from integrating with emergency medicine services to usual primary care services. Using our early case site visits as examples, we were able to map out constructs (from the glossary of terms, online supplementary data 6) that influenced service function. We grouped these at the wider system, department and individual clinician levels,^20^ to develop a conceptual summary of constructs that influence the function of these services (figure 2). This could be used to consider whether the constructs aligned with the form adopted.^8^ For example, whether the constructs of function for an ‘INSIDE integrated’ model align with an emergency medicine service or if the constructs of function for an ‘INSIDE parallel’ or ‘OUTSIDE on site’ model align with usual primary care services.

**Figure 3:**
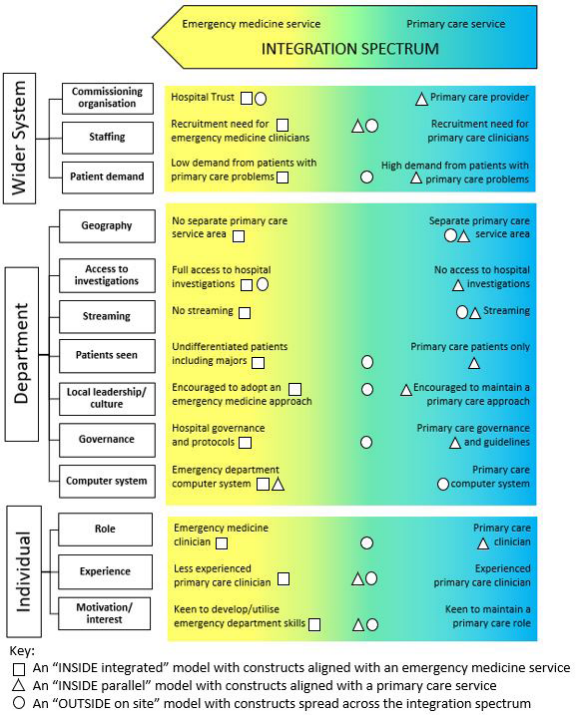
The taxonomy applied to case site examples

Each respective construct may influence the overall function of the service including: the demographic and morbidity profile of the local population; demand from patients with primary care type problems; staff recruitment needs; department-level clinical leadership and culture; contractual and payment arrangements; and the skill mix and personal interest of the GPs and other primary care health professionals. Staff may be deployed in more than one mode and constructs may also vary according to service pressures, time of day, staff availability and other influences. The taxonomy as applied to some case site examples is shown in figure 3.

### Emergency medicine service

Constructs that contribute to primary care staff adopting an emergency clinician role and the primary care service developing towards and integrating with the emergency medicine service include difficulties in recruiting emergency medicine clinicians and low demand from patients with primary care type problems. Other factors include: primary care clinicians with less experience in primary care and greater interests in emergency medicine; employment by the NHS Hospital Trust; financial and contractual models that favour emergency department service provision; encouragement by local leaders to follow Trust protocols and governance systems; and the primary care service located in the same area as emergency department clinicians with full access to hospital investigations and seeing unselected patients.

The ‘INSIDE integrated’ service represented in figure 3 is a small rural hospital with a lack of demand for patients with primary care problems and a recruitment need for emergency department clinicians. GPs with an interest in emergency medicine, keen to develop their skills, are employed by the Hospital Trust. They work in the same area as the emergency department clinicians seeing a full range of undifferentiated patients with full access to acute diagnostics; no formal streaming process is in operation. They are supported by the emergency department consultants and expected to follow the emergency department guidelines and governance systems. Here, constructs of function align with an emergency medicine service.

### Primary care service

Constructs that encourage a primary care service include employment by a primary care provider and primary care guidelines, governance and clinical record computer systems. Other factors include: a high demand from patients with primary care type problems; patients with primary care problems streamed directly to the primary care service; primary care clinicians working in a separate area with limited access to hospital investigations (or advised not to use); contractual and payment models that incentivise the delivery of a primary care service; and local leadership encouraging the practitioners to treat patients as they would in a primary care setting. An additional influence is from the primary care clinicians themselves being keen to maintain primary care roles.

The ‘INSIDE parallel’ model represented in figure 3 is in a large town. The primary care service was previously a separate distinct service across the road but has now been incorporated into a separate area within the emergency department. GPs are commissioned by a primary care provider, encouraged not to use acute investigations and maintain a primary care role. Patients with primary care problems are streamed to the service; there was reported to be high demand. Here, constructs of function align with a primary care service.

### Variation across the integration spectrum

Some sites had a less consistent alignment of constructs of function with the service model form. For example, the ‘OUTSIDE on site’ model represented in figure 3 is on a city hospital site, 100 metres from the emergency department entrance. Primary care clinicians are employed by the Hospital Trust and emergency department advanced nurse practitioners also staff the unit, following emergency department protocols and policy; there was not reported to be any specific emergency medicine or primary care recruitment issues. Patients with primary care problems and some minor injuries are streamed from the emergency department; demand can fluctuate. Clinicians adopt a different approach to the out-of-hours primary care practitioners that work out in the same area, using emergency department acute investigations if needed. Here, constructs of function are spread across the spectrum of integration.

### Stakeholder feedback

The taxonomy was iterated following discussions with the co-applicant groups from both studies in August and September 2018 (18 members from Cardiff, 17 from the University of the West of England) and with the teams’ steering committees in October 2018. It was presented, as applied to some case study sites, to 64 stakeholders (largely commissioners and multidisciplinary service providers) at a further event in November 2018. Discussions with commissioners and service providers at this stakeholder event highlighted the complex adaptive (and evolving) interaction between primary care and emergency department services. Stakeholders reported that the taxonomy and integration spectrum was useful to identify whether constructs of function within their departments were consistent with the form of service provided and whether some constructs may be modifiable to enhance this alignment to achieve the intended aims. They envisaged that it could also support discussions about the longevity and sustainability of their current services and incremental benefit of changing the model.

### Limitations

We recognise that while we have tried to capture the most common influences on function, other contextual factors (for example rurality, additional local services) may also influence how models operate. We are not yet able to describe which constructs or combinations of constructs have the strongest influence on function, and this may vary by location and context. We focused on the UK where many services are in a state of change, making generalisation difficult. Further research is necessary to validate the taxonomy with additional sites and stakeholders and to determine whether the taxonomy is valid in other countries and healthcare systems.

A strength of this work is that the collaboration between two study teams meant that we could use multiple data sources to gain information about 62% of Type 1 emergency departments in England and Wales. Little information was available about the non-responders to assess response bias. However, there were similar application rates for capital bid funding in survey responders and non-responders suggesting a representative sample. Despite no formal consensus exercise, we had strong stakeholder participation including representatives from policy and commissioning groups, service leaders and providers, GPs and advanced nurse practitioners, public contributors and academic teams.

### Summary

We used findings from a literature review, a national survey, staff interviews, other data sources and discussions with stakeholders to develop a taxonomy based on a conceptual spectrum of integration—identifying constructs that influence whether primary care service models in or alongside emergency departments function closer to an emergency medicine service or to usual primary care. We have also simplified the classification for the forms they adopt, INSIDE (integrated or parallel models) or OUTSIDE (on or off site) the emergency department, to provide a framework for further research and enable comparison of models and outcomes.

Consistency of terminology and classification of models in practice is essential for rigorous research to evaluate these service models for patient-level health and experience outcomes, health economics and wider system implications. Only then can the evidence base inform policy and national guidelines. The taxonomy will now be implemented in the two UK NIHR-funded studies, purposefully selecting study sites that exemplify the different model types, to evaluate their effectiveness and inform decision-making and future policy.^13 14^

Commissioners and service providers can consider these constructs when characterising and designing services, depending on the needs of the local population, and whether policy and clinical leads require a primary care or emergency medicine service.

10.1136/emermed-2018-208305.supp5 Supplementary file 5
