# Supplementary material for: Taxonomy of the form and function of primary care services in or alongside emergency departments: concepts paper
Source: Emerg Med J. 2019 Sep 7;36(10):625–30. doi: 10.1136/emermed-2018-208305 (PMC6837280; doi:10.1136/emermed-2018-208305)
Supplement: Supplementary file 1 [file emermed-2018-208305supp001.pdf]

GP ED

GPs in Emergency Departments

GPs in Emergency Departments Study

CARDIFF UNIVERSITY PRIFYSGOL CAERDYDD

UWE Bristol University of the West of England

We are conducting a national study to evaluate the **clinical and cost effectiveness** of using GPs in or alongside Emergency Departments. We aim to address the key policy questions of where and how the greatest value can be delivered by using GPs in the ED setting. The study is funded by NIHR's Health Services and Delivery Research Programme.

We are inviting **Clinical Directors** of Type 1 EDs in England and Wales to complete this survey. We would like to hear from you **whether or not** there are GPs working at your ED. If you are the Clinical Director for more than one ED, please fill out a separate survey for **each** of your EDs.

The survey should take approximately 15 minutes to complete. We appreciate you taking the time to complete the survey and contributing to this research.

By completing this survey, I am consenting to take part in this study. I understand my data will be held securely and I have a right to withdraw from this study at any time. I understand that when this information is no longer required for this purpose, official Cardiff University procedure will be followed to dispose of my data ✱ *Required*

- ◯ I agree
- ◯ I disagree

Decline participation

You have indicated that you do not wish to complete this survey. If you have any further questions or comments about the study, please do not hesitate to contact the research team on **GP-EDStudy@cardiff.ac.uk**. Many thanks

- End survey now

Contact Details

Name of Emergency Department (ED) ✱ *Required*

Your name ✱ *Required*

Job title

Contact telephone number

Please enter a valid phone number.

E-mail address

Please enter a valid email address.

GP Out-of-Hours Service

Do you currently have a GP Out-of-hours (OOH) service on your hospital site? ✱ *Required*

- ◯ Yes
- ◯ No

GP Out-of-Hours Service

Can ED staff (including receptionists, nurses, doctors) re-direct patients presenting to the emergency department to the GP OOH service? ✱ *Required*

- Yes
- No

Redirecting patients from ED to GP Out-of-Hours

How do staff re-direct patients to the GP OOH service?

- ☐ Staff make a GP OOH appointment for the patient
- ☐ Staff give patients the telephone number of GP OOH to make an appointment themselves
- ☐ Staff direct patients to the GP OOH, where patients can walk in and make an appointment themselves
- ☐ Other

If you selected Other, please specify:

Selecting patients to be seen by GP OOH

Who selects patients to be re-directed to the GP Out-of-Hours service? *(Select all that apply)*

- ☐ ED Receptionist
- ☐ ED nurse
- ☐ ED doctor
- ☐ GP working in the ED
- ☐ GP working in GP Out-of-hours self-selects
- ☐ Primary care nurse working in GP Out-of hours
- ☐ Paramedics select patients brought in by ambulance to be seen by GP OOH
- ☐ Other

If you selected Other, please specify:

When selecting patients, do they: *(select all that apply)*

- ☐ Use locally-developed inclusion / exclusion criteria
- ☐ Use a national tool e.g. Manchester triage system (please specify below)
- ☐ Use clinical judgement
- ☐ Other

If you selected Other, please specify:

If a national tool is used in selecting patients to be seen by GP OOH, please specify which one:

GP OOH - Patient volume

Approximately what proportion of patients presenting to the ED are **re-directed to the GP Out-of-Hours service**, during an average week?

- Less than 1%
- 2 - 4%
- 5 - 7%
- 8 - 10%
- 11 - 15%
- 16 - 20%
- More than 20%
- Don't know

GPs in ED

Do you currently have GPs working in any other way, within or alongside your Emergency Department? *We realise that GPs are being used in many different models - please select Yes if GPs are involved in delivering your acute care service* ***in any way,*** *apart from in GP OOH services.*

- Yes
- No

EDs without a GP service

Have you ever had GPs working in or alongside your ED in the past? ✱ *Required*

- ○ Yes
- ○ No

Previous GP service - Timing

If your ED has previously used GPs on multiple occasions in different ways, please answer these next questions about the **most recent** time GPs were used

When did they start? *(If exact dates not known, please give an estimate)*

Dates need to be in the format 'DD/MM/YYYY', for example 27/03/1980.

(dd/mm/yyyy)

When did they stop? *(If exact dates not known, please give an estimate)*

Dates need to be in the format 'DD/MM/YYYY', for example 27/03/1980.

(dd/mm/yyyy)

Don't know

- I don't know when the GP service was running

Previous GP service - Location

Where were the GPs working? *(please select one)*

- Within the ED alongside ED clinicians
- Within the ED but as a separate unit
- Adjacent to the ED but common entrance
- Adjacent to the ED but separate entrance
- On hospital site but separate from the ED
- Don't know
- Other

If you selected Other, please specify:

Previous GP Service - Model

Which patients did the GPs see? *(select all that apply)*

- ☐ Patients with primary care problems (cases that would frequently present to general practice, that all GPs would feel confident in treating)
- ☐ Low acuity patients that may have included minor trauma
- ☐ Only specific patient groups e.g paediatrics, frail elderly
- ☐ Undifferentiated patients that presented to ED (i.e. the same case mix as ED clinicians)
- ☐ Patients at the ED front-door, directing them to the most appropriate healthcare provider e.g. into the ED, back to their own GP, pharmacist, optician, dentist etc
- ☐ Patients at the ED front-door, directing them to the most appropriate area or clinician within the ED
- ☐ Don't know
- ☐ Other

If you selected Other, please specify:

Previous GP service - Funding

Which option best describes **provision** and **funding** of the GP service that used to be in place? *(please select one)*

- The hospital trust provided the service
- A private company provided the service, paid for by the CCG or Health Board
- A local GP group provided the service, paid for by the CCG or Health Board
- The same company as provided the local Out-of-Hours service provided the GP in ED service, paid for by the CCG or Health Board
- Don't know
- Other

If you selected Other, please specify, including who paid for the service:

Plans for a GP service

Are there any **plans** to implement a new model of using GPs in or alongside your ED **within the next 12 months**? ✱
Required

- Yes
- No

Planned GP service

When are you planning to implement the new GP service?

- Within the next 3 months
- Within the next 6 months
- Within the next 9 months
- Within the next 12 months

Is your ED making a 2017 capital bid following the chancellor’s budget announcement in March?

- Yes
- No

Planned GP Service - Location

Where will the GPs work? *(please select one)*

- Within the ED alongside ED clinicians
- Within the ED but in a separate unit
- Adjacent to the ED but common entrance
- Adjacent to the ED but separate entrance
- On hospital site but separate from the ED
- Don't know
- Other

If you selected Other, please specify:

Planned GP Service - Hours

What **hours** will GPs work in or alongside your ED? *(select all that apply)*

	Daytimes	Evenings	Nights
Weekdays	☐	☐	☐
Weekends	☐	☐	☐

Planned GP Service - Primary care workforce

What **sort of primary care clinicians** will be used within or alongside the ED? *(select all that apply)*

- ☐ Salaried GPs (fixed shifts per week)
- ☐ Sessional/locum GPs (ad-hoc shifts)
- ☐ Advanced nurse practitioners (fixed shifts)
- ☐ Advanced nurse practitioners (ad-hoc shifts)
- ☐ Other nurses (fixed shifts)
- ☐ Other nurses (ad-hoc shifts)
- ☐ Don't know
- ☐ Other

If you selected Other, please specify:

Planned GP Service - Model

Which patients will the GPs see? *(select all that apply)*

- ☐ Patients with primary care problems (cases that would frequently present to a general practice, that all GPs would feel confident in treating)
- ☐ Low acuity patients that may include minor trauma
- ☐ Only specific patient groups e.g. paediatrics, frail elderly
- ☐ Undifferentiated patients that present to ED (i.e. the same case mix as ED clinicians)
- ☐ Patients at the ED front-door, directing them to the most appropriate healthcare provider e.g. into the ED, back to their own GP, pharmacist, optician, dentist etc.
- ☐ Patients at the ED front-door, directing them to the most appropriate area or clinician within the ED
- ☐ Don't know
- ☐ Other

If you selected Other, please specify:

Planned GP Service - Funding

Which option best describes **provision** and **funding** of the planned GP service? *(please select one)*

- The hospital trust will provide the service
- A private company will provide the service, paid for by the CCG or Health Board
- A local GP group will provide the service, paid for by the CCG or Health Board
- The same company as provides the local Out-of-Hours service will provide the service, paid for by the CCG or Health Board
- Don't know
- Other

If you selected Other, please specify, including who will pay for the service:

Setting up a GP service

What are the **barriers** to setting up a GP service in your ED? *(select all that apply)*

- ☐ Lack of funding
- ☐ Difficulty sourcing primary care staff
- ☐ Difficulty sourcing facilities
- ☐ Not perceived as beneficial
- ☐ Governance issues (quality improvement, accountability)
- ☐ Training concerns
- ☐ Other

If you selected Other, please specify:

What would better **enable** your ED to set up a GP service?

Marker Conditions

Can you think of any examples of presenting complaints or conditions which may be **managed better** by GPs compared to traditional ED staff?

More info

1.	
2.	
3.	

Can you think of any examples of presenting complaints or conditions which may be **managed less well** by GPs compared to traditional ED staff?

More info

1.	
2.	
3.	

Patient volume

Approximately how many patients presented to your Emergency Department in the last 12 months?

Aims of GP Service

What were the **aims** of introducing GPs in or alongside your ED? (*Select all that apply)*

- ☐ Reduce ED patient volume
- ☐ Reduce ED waiting times
- ☐ Reduce hospital admissions
- ☐ Better use of available ED resources
- ☐ Improve patient experience
- ☐ Improve quality of care given to certain types of cases / patients
- ☐ To 'educate' patients by sending them to the right place
- ☐ Cost saving
- ☐ National (or other) directive
- ☐ I don't know what the aims were
- ☐ Other

If you selected Other, please specify:

Aims of GP Service

Does the GP service achieve these aims **in practice**? *(only answer for those aims selected in the previous question)*

Please don't select more than 1 answer(s) per row.

	All of the time	Most of the time	Sometimes	Rarely	Never	Don't know
Reduce ED patient volume	☐	☐	☐	☐	☐	☐
Reduce ED waiting times	☐	☐	☐	☐	☐	☐
Reduce hospital admissions	☐	☐	☐	☐	☐	☐
Better use of available ED resources	☐	☐	☐	☐	☐	☐
Improve patient experience	☐	☐	☐	☐	☐	☐
Improve quality of care given to certain types of cases / patients	☐	☐	☐	☐	☐	☐
To 'educate' patients by sending them to the right place	☐	☐	☐	☐	☐	☐
Cost saving	☐	☐	☐	☐	☐	☐

Can you suggest any reasons why the aims were, or were not met?

Setting up your GP service

Approximately when did GPs **first start** working in or alongside your ED?

Dates need to be in the format 'DD/MM/YYYY', for example 27/03/1980.

(dd/mm/yyyy)

What factors do you think **enabled** setting up of the GP service?

Were there any **barriers** to setting up in the GP service? If so, what were they?

Changes to your GP service

Have any **changes** been made to the model since it was first introduced?

- Yes
- No
- Don't know

If Yes, when and how?

Are there any plans to implement a **new model** of using GPs in or alongside your ED within the next 12 months?

- No
- Yes, within 3 months
- Yes, within 6 months
- Yes, within 12 months

If Yes, what changes are planned? *(Please specify)*

Is your ED making a 2017 capital bid following the chancellor’s budget announcement in March?

- Yes
- No

Current GP Service - Location

Where do the GPs work? *(Please select one)*

- Within the ED alongside ED clinicians
- Within the ED but in a separate unit
- Adjacent to the ED but common entrance
- Adjacent to the ED but separate entrance
- On hospital site but separate from the ED
- Other

If you selected Other, please specify:

Current GP Service - Hours

What **hours** do GPs work in or alongside your ED? *(Select all that apply)*

	Daytimes	Evenings	Nights
Weekdays	☐	☐	☐
Weekends	☐	☐	☐

In an average week, what is the **total aggregate time** GPs spend working in or alongside your ED?

More info

- Less than 9 hours
- 9 - 16 hours
- 17 - 24 hours
- 25 - 32 hours
- 33 - 40 hours
- 41 - 48 hours
- 49 - 56 hours
- 57 - 64 hours
- 65 - 72 hours
- 73 - 80 hours
- More than 80 hours
- Don't know

Current GP Service - Primary care workforce

Approximately **how many GPs** work within or alongside your ED each week?

- 1 - 3
- 4 - 6
- 7 - 9
- 10 - 12
- 13 - 15
- More than 15
- Don't know

Approximately **how many GPs** work **two or more shifts** each week?

- 1 - 3
- 4 - 6
- 7 - 9
- 10 - 12
- 13 - 15
- More than 15
- Don't know

What **sort of primary care clinicians** are used within or alongside the ED? *(Select all that apply)*

- [ ] Salaried GPs (fixed shifts per week)
- [ ] Sessional/locum GPs (ad-hoc shifts)
- [ ] Advanced nurse practitioners (fixed shifts)
- [ ] Advanced nurse practitioners (ad-hoc shifts)
- [ ] Other nurses (fixed shifts)
- [ ] Other nurses (ad-hoc shifts)
- [ ] Other

If you selected Other, please specify:

Current GP Service - Model

Which patients do the GPs see? *(Select all that apply)*

- ☐ Patients with primary care problems (cases that would frequently present to a general practice, that all GPs would feel confident in treating)
- ☐ Low acuity patients that may include minor trauma
- ☐ Only specific patient groups e.g. paediatrics, frail elderly
- ☐ Undifferentiated patients that present to ED (i.e. the same case mix as ED clinicians)
- ☐ Patients at the ED front-door, directing them to the most appropriate healthcare provider e.g. into the ED, back to their own GP, pharmacist, optician, dentist etc.
- ☐ Patients at the ED front-door, directing them to the most appropriate area or clinician within the ED
- ☐ Other

If you selected Other, please specify:

Current GP Service - Selecting patients to be seen by a GP

Who selects patients to be seen by a GP? *(Select all that apply)*

- ☐ Patient self-refers, no triage system in place
- ☐ Receptionist
- ☐ ED nurse
- ☐ ED doctor
- ☐ GP self-selects
- ☐ Primary care nurse
- ☐ Telephone triage primary care service make appointment
- ☐ Paramedics select patients brought in by ambulance to the GP
- ☐ Other

If you selected Other, please specify:

When selecting patients, do they: *(Select all that apply)*

- ☐ Use locally-developed inclusion / exclusion criteria
- ☐ Use a national tool e.g. Manchester triage system (please specify below)
- ☐ Use clinical judgement
- ☐ Other

If you selected Other, please specify:

If a national tool is used in selecting patients to be seen by a GP, please specify which one:

Current GP Service - Patient volume

Appoximately how many patients **presented to your Emergency Department in the last 12 months**?

Approximately what **percentage of all patients** presenting to your ED are **seen by GPs or other primary care staff** (e.g. nurses)?

Don't know

- ○ I don't know what proportion of patients are seen by primary care staff

Current GP Service - GPs access to investigations

Which **investigations** do the GPs have access to? *(select all that apply)*

- ☐ Blood tests
- ☐ Other laboratory tests such as microscopy and culture
- ☐ ECGs
- ☐ Plain X-rays
- ☐ Other imaging including CT, MRI, ultrasound, contrast studies
- ☐ Near patient testing e.g. point of care CRP
- ☐ None of the above

Marker Conditions

Can you think of any examples of presenting complaints or conditions which may be **managed better** by GPs compared to traditional ED staff?

More info

1.	
2.	
3.	

Can you think of any examples of presenting complaints or conditions which may be **managed less well** by GPs compared to traditional ED staff?

More info

1.	
2.	
3.	

Current GP Service - Funding

Which option best describes **provision** and **funding** of the GP service? *(Please select one)*

- ○ The hospital trust provides the service
- ○ A private company provides the service, paid for by the CCG or Health Board
- ○ A local GP group provides the service, paid for by the CCG or Health Board
- ○ The same company as provides the local Out-of-Hours service provides the GP in ED service, paid for by the CCG or Health Board
- ○ Don't know
- ○ Other

If you selected Other, please specify, including who paid for the service:

Current GP Service - Training and Governance

Which organisation has responsibility for **induction and training** for the GPs operating within or alongside the ED? *(Please select one)*

- The hospital
- The primary care provider organisation
- An organisation that is a legal entity established for the purpose
- Shared responsibility between the hospital and primary care provider organisation
- Don't know
- Other

If you selected Other, please specify:

Which organisation has responsibility for **clinical audit and governance** for GPs operating within or alongside the ED? *(Please select one)*

- The hospital
- The primary care provider organisation
- An organisation that is a legal entity established for the purpose
- Split accountability and governance arrangements between the hospital and primary care provider organisation
- Don't know
- Other

If you selected Other, please specify:

Co-located GP Services

If you have a co-located GP service and could not answer all of the questions about their service, please provide the name of the **GP Provider** so that we can contact them for further information

Contact name *Optional*

Job title

Address

Telephone number

Please enter a valid phone number.

E-mail address

Please enter a valid email address.

End of Survey

Thank you for taking the time to complete this survey.

We may get in touch to clarify any information if needed.

Any further comments are welcome

Thank you

Your contribution to this research is greatly appreciated
