# Supplementary material for: Taxonomy of the form and function of primary care services in or alongside emergency departments: concepts paper
Source: Emerg Med J. 2019 Sep 7;36(10):625–30. doi: 10.1136/emermed-2018-208305 (PMC6837280; doi:10.1136/emermed-2018-208305)
Supplement: Supplementary file 3 [file emermed-2018-208305supp003.pdf]

GPED Interview Guide

ED context:
- How many new adult patients does your ED see each year?
- How many new paediatric patients does your ED see each year?

What model of working with GPs/primary care operates in your ED currently (if any)?

Tell us about any GPED model you are planning to implement:
Can you tell us the background to that decision?
- What are you hoping to achieve
- What discussions took place
- What options were considered
- What major factors impacted on decision making

How is it different from the model you have in place now (is it clearly distinct)?
- Structural requirements for proposed model
- Organisational requirements for proposed model
- How will changes (if any) be achieved
- Timetable for change (date)

Do you think this model makes sense/is the right thing for your department?

Do you think staff value the proposed model of service provision?

What do you think the impact will be to your department on:
- Performance (4 hours, hospital admission rate)
- Staff (which staff in particular, in what ways)
- Division of labour
- Interaction between different professional groups
- Resources
- Consultations with patients

Will staff require additional training before implementation?
- Which staff and what training in planned/available

How will you judge the success/impact of the new mode of service delivery?
- What data might be available for research purposes
- Mechanism for staff feedback about the intervention

What are your thoughts on the decision to fund these models of service delivery?
- Does the idea of GPs in ED make sense in general

Any other comments to add about GPED?
