# Supplementary material for: Taxonomy of the form and function of primary care services in or alongside emergency departments: concepts paper
Source: Emerg Med J. 2019 Sep 7;36(10):625–30. doi: 10.1136/emermed-2018-208305 (PMC6837280; doi:10.1136/emermed-2018-208305)

Data sources for information about Type 1 emergency departments and associated primary care services in England and Wales in 2017-18

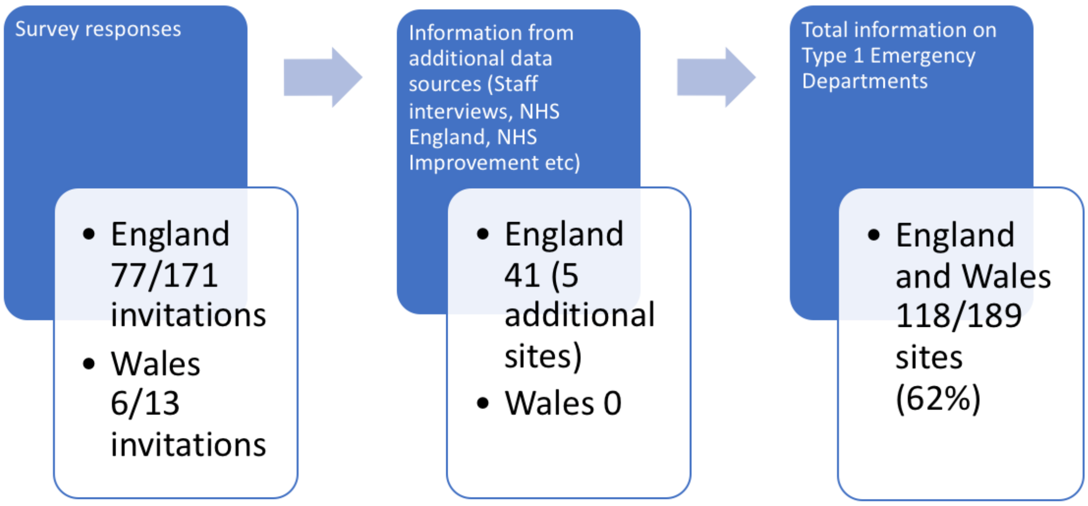

Supplement: Supplementary file 4 [file emermed-2018-208305supp004.pdf]
