# Supplementary material for: Taxonomy of the form and function of primary care services in or alongside emergency departments: concepts paper
Source: Emerg Med J. 2019 Sep 7;36(10):625–30. doi: 10.1136/emermed-2018-208305 (PMC6837280; doi:10.1136/emermed-2018-208305)
Supplement: Supplementary file 6 [file emermed-2018-208305supp006.pdf]

Supplementary data 1: Glossary of terms presented at the first stakeholder conference

**Constructs**		**Options**	**Description of options**
1. Location	Where the GP service is located in relation to the ED	Adjacent	GP service is located next to ED but has a separate entrance
		Co-located	GP service is located next to the ED and has a common entrance
		Embedded	GP service operates in a separate unit within the ED
		Integrated	GPs work in the ED alongside ED clinicians
2. Redirection	Appropriate patients are redirected **out of the ED**	Signposted	The patient is signposted to a walk-in GP service
		Appointment made	An in-hours GP appointment or GP OOH is made for the patient
		No appointment made	It is the patient's responsibility to make a GP appointment
	Staff member redirecting	Receptionist	No clinical training
		ED nurse	ED staff nurse
		ED nurse practitioner	To include emergency and advanced nurse practitioners
		ED clinician	To include ED consultants, staff grade and doctors in training
		GP	To include salaried and locum GPs
		Primary care nurse practitioner	To include primary care and out-of-hours nurse practitioners
		Primary care nurses	Primary care practice and out-of-hours nurses
3. Streaming	Patients are directed **into the ED** to the most appropriate healthcare provider	Emergency department	The patient has an urgent/ life threatening condition requiring ED level care
		Primary care	The patient's condition meets the criteria for the primary care stream
		Minor injuries	The patient has a minor injury
		See and treat	The patient is seen and treated by the Streamer
		No streaming system	ED Clinicians and GPs self-select their own patients

Supplementary data 1: Glossary of terms presented at the first stakeholder conference

	Staff member streaming	Receptionist	No clinical training
		ED nurse	ED staff nurse
		ED nurse practitioner	To include emergency and advanced nurse practitioners
		ED clinician	To include ED consultants, staff grade and doctors in training
		GP	To include salaried and locum GPs
		Primary care nurse practitioner	To include primary care and out-of-hours nurse practitioners
		Primary care nurse	Primary care practice and out-of-hours nurse
	Streaming/ triage guidance	Manchester triage system	A validated and widely used triage system
		Locally developed criteria	Locally developed or adapted criteria
		Other	Alternative guidance
4. GP role	The role the GP is expected to adopt in the ED setting	Traditional GP role	Managing patients using the same approach taken in the primary care setting – minimal use of acute investigations
		Extended GP role	The GP takes on additional tasks, which might not be part of routine general practice (e.g. seeing patients with minor injuries) or focusses on specific groups of patients in the ED (e.g. paediatrics, frail elderly)
		Gatekeeper role	Redirecting appropriate primary care type patients back into the community for treatment
		ED clinician role	The GP acts as 'another pair of hands' as another ED clinician
	Patients seen by the GP	Primary care type problems	A complaint that the average GP in the average GP surgery would be able to manage
		Non-urgent problems	Non-urgent problems including minor injury
		Special patient groups	Special groups of patients i.e. paediatrics, frail elderly
		Undifferentiated patients	Same case mix as ED clinicians
	GP access to investigations	GP tests only	Investigations found in the average GP surgery i.e. urine dipstix, ECG
		GP tests and plain X rays	GP tests and plain X rays
		Hospital tests	Acute blood tests and radiology i.e. Plain X rays and CT scans

Supplementary data 1: Glossary of terms presented at the first stakeholder conference

5. GP service provision	The organisation responsible for providing the GP service	Hospital	The hospital is providing the GP service, directly employs GPs
		Private GP provider	A private company provides the GP service
		Local GP group	A local GP group provides the GP service
	Clinicians employed	GPs	To include salaried and locum GPs
		Primary care nurse practitioners	To include primary care and out-of-hours nurse practitioners
		Primary care nurses	Primary care practice and out-of-hours nurses
	Employment basis	Fixed shifts	Staff who have a set number of hours/shifts on a regular basis
		Ad hoc shifts	Staff cover shifts based on the needs of the department
	Service coverage	7 days a week	Usual GP service coverage per week (actual coverage rather than planned)
		5-6 days a week	
		3-4 days a week	
		1-2 days a week	
	Shift pattern	Day shift	Usual shift pattern covered (can select multiple options)
		Evening shift	
		Night shift	
		Weekends	
	Percentage patients presenting to the ED seen by GPs	<5%	Approximate number of patients presenting to the ED seen by the GP service annually
		6-10%	
		11-30%	
		>31 %	

Supplementary data 1: Glossary of terms presented at the first stakeholder conference

6. Hospital demographics	Volume of patients attending the ED annually	Less than 50,000	
		51,000-100,000	
		101,000-150,000	
	Geographical description	Rural	Located outside large towns and cities
		Urban	Located in a large town or city
	Teaching hospital	Teaching	Affiliated with a medical school and has students
		Non-teaching	Not affiliated with a medical school and has no students

Key:

GP	General Practitioner
ED	Emergency Department
GP OOH	GP out-of-hours service
